# Fleshy or dry: transcriptome analyses reveal the genetic mechanisms underlying bract development in *Ephedra*

**DOI:** 10.1186/s13227-022-00195-4

**Published:** 2022-04-27

**Authors:** Cecilia Zumajo-Cardona, Barbara A. Ambrose

**Affiliations:** 1 New York Botanical Garden, Bronx, NY USA; 2 The Graduate Center, City University of New York, New York, NY USA

**Keywords:** Convergent evolution, Gnetales, Integument, Model organisms, Ovule, RNAseq, Seed development

## Abstract

**Background:**

Gnetales have a key phylogenetic position in the evolution of seed plants. Among the Gnetales, there is an extraordinary morphological diversity of seeds, the genus *Ephedra*, in particular, exhibits fleshy, coriaceous or winged (dry) seeds. Despite this striking diversity, its underlying genetic mechanisms remain poorly understood due to the limited studies in gymnosperms. Expanding the genomic and developmental data from gymnosperms contributes to a better understanding of seed evolution and development.

**Results:**

We performed transcriptome analyses on different plant tissues of two *Ephedra* species with different seed morphologies. Anatomical observations in early developing ovules, show that differences in the seed morphologies are established early in their development. The transcriptomic analyses in dry-seeded *Ephedra californica* and fleshy-seeded *Ephedra antisyphilitica*, allowed us to identify the major differences between the differentially expressed genes in these species. We detected several genes known to be involved in fruit ripening as upregulated in the fleshy seed of *Ephedra antisyphilitica*.

**Conclusions:**

This study allowed us to determine the differentially expressed genes involved in seed development of two *Ephedra* species. Furthermore, the results of this study of seeds with the enigmatic morphology in *Ephedra californica* and *Ephedra antisyphilitica*, allowed us to corroborate the hypothesis which suggest that the extra envelopes covering the seeds of Gnetales are not genetically similar to integument. Our results highlight the importance of carrying out studies on less explored species such as gymnosperms, to gain a better understanding of the evolutionary history of plants.

**Supplementary Information:**

The online version contains supplementary material available at 10.1186/s13227-022-00195-4.

## Background

Gnetales is one of the most extraordinary lineages of seed plants (i.e., Cycadales, Ginkgoales, Coniferales and angiosperms). With three genera within Gnetales, *Ephedra* is sister to *Gnetum* and *Welwitschia*, and it is the most diverse among the three, distributed in the desert regions worldwide [1–3]. The morphology of *Ephedra* is very peculiar; it is a small shrub, climber or small tree; with green stems, scale-like leaves, and strobili with 2–8 pairs of decussate bracts, where the proximal are sterile and the distal bracts are fertile. *Ephedra* is usually dioecious, with unisexual cones born in the axils of bracts. Within the fertile bracts of the ovulate strobili, there are one to three ovules [4, 5], while in the staminate strobili, the antherophore is stalked consisting of two fused microsporophylls bearing 2–8 stalked or sessile synangia [2, 6, 7]. In the ovulate strobili, each ovule is surrounded by one to two additional bracts (also called envelopes) and the integument [4, 5]. The integument forms a micropylar projection which produces a pollination droplet; the integument will become the seed coat or testa [3–5].

**Fig. 1:**
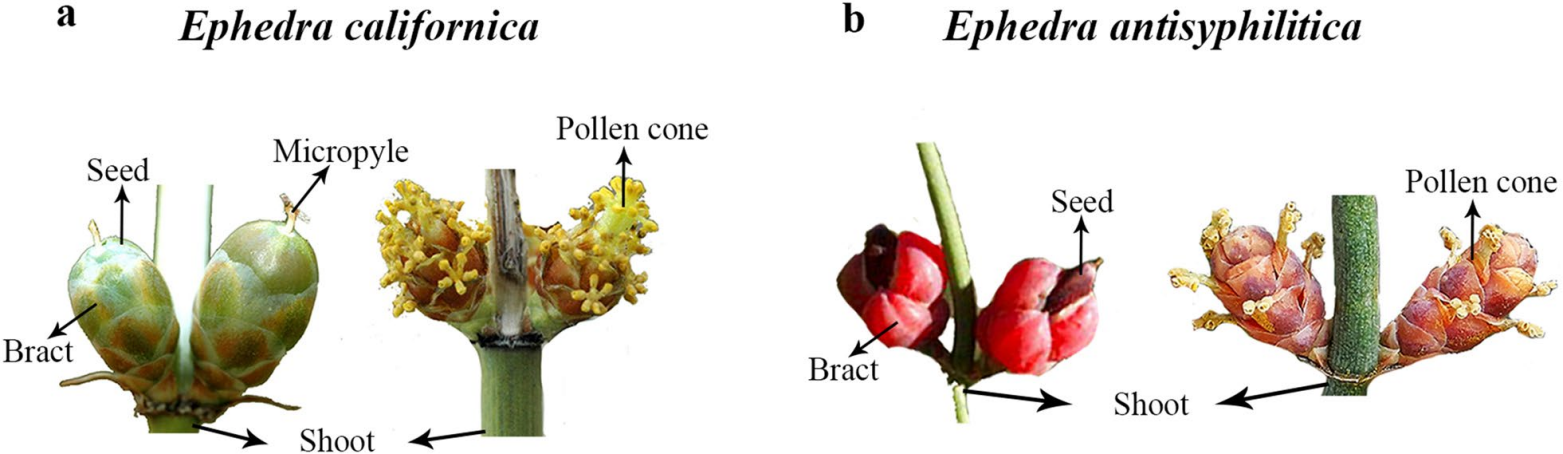
Morphology of the *Ephedra* species used in this study. **a** *Ephedra californica* ovules (left) with dry seeds and pollen cones (right). **b** *Ephedra antisyphilitica* ovules (left) with fleshy seeds and pollen cones (right)

Between *Ephedra* species, there is morphological variation in these additional bracts surrounding the seeds that may impact seed dispersal mechanisms and are the focus of this study (Fig. 1). The fleshy bracts attract birds and lizards that ensure the dispersal of the seeds [8]; the dry winged bracts ensure wind dispersal; and seeds with membranous bracts are dispersed by rodents [3, 9–11]. The bracts associated with the ovule, have been described by many authors as integuments, which for Eames [4] is ‘unfortunate’, since this term suggests a homology with the outer integument of angiosperms; adding that there is no morpho-anatomical evidence suggesting that the bracteoles are in fact integuments [3, 4, 12]. Moreover, expression analyses in *Gnetum gnemon*, show that the known angiosperm integument developmental genes are not expressed in the bracts (also called envelopes), suggesting that genetically the extra-bracts of *Gnetum* are not integuments [13].

The study of fleshy seeds in gymnosperms is important to understand the genetic basis of fleshiness in general. Questions such as the possible convergent evolution between fleshy seeds and fruits, or the evolution of fleshiness itself remain to be answered. Several MADS-box genes known to be involved in ovule development have been studied in gymnosperms with fleshy seeds such as *Cycas*, *Ginkgo*, *Taxus* and *Gnetum* [14–17]. The evolution of MADS-box genes has been thoroughly studied across land plants; specifically, *AG*, *AGL6* and *B-sister* genes have been reported across seed plants [16, 18–22]. In angiosperms, genes such as *AGAMOUS* (*AG*) and *SEPALLATA* (*SEP*) are known to be involved in carpel and fruit development in angiosperms [23–25]. In the gymnosperms, such as *Ginkgo* and *Taxus*, *AG-like* homologs are known to be involved in the development of reproductive structures: ovules and pollen cones [15, 16, 19, 22]. *SEP* genes, on the other hand, do not seem to have direct homologs in gymnosperms, but its sister clade *AGL6* does [21, 26]. The *AGL6* homologs, *GbMADS1* and *GbMADS8*, have been reported to be putatively involved in the development of the integument in *Ginkgo* as well as in the ovule and aril in *Taxus baccata* [16]. However, no expression was found in the ovules of *Gnetum gnemon* [19, 26].

In addition there are two *B-sister* genes, also belonging to the large MADS-box transcription factor family [27–29], specific to Brassicaceae: *TRANSPARENT TESTA 16* (*TT16*) and *GORDITA* (*GOA*), with pre-duplication genes identified in seed plants [20, 29]. *B-sister* genes are involved in the correct differentiation of ovule/seed but also in fruit development [28–33] and are also found expressed in the ovule of *Ginkgo biloba* [17]. However, the putative function of *B-sister* genes in ovule development in gymnosperms seems to be more intricate since no expression is detected in *Taxus baccata* [17].

As *AG, AGL6* and *B-sister* genes are known to be involved in ovule development in seed plants and have been found expressed in the fleshy tissues of some gymnosperms, the study of these genes in *Ephedra* species with different bract morphologies, dry or fleshy provides an excellent framework for a better understanding of the role these MADS-box genes may play in ovule development and ultimately, find out whether they are functionally conserved across seed plants.

The phylogenetic position of Gnetales is key to understanding the evolution of seed plants and is still a subject of debate. Its morphological traits places Gnetales as the sister group of angiosperms, which is not in agreement with the molecular data: moreover, an emerging consensus places them nested within Coniferales [34–40]. It should be noted that *Ephedra* is the only group of gymnosperms that includes small species with a relatively rapid transition to the reproductive stage, i.e., *Ephedra monosperma* is a small shrub, up to 20 cm long, with a pair of fleshy bracts surrounding the seed. In addition, *Ephedra gerardiana* has been reported to take about 4 months to produce viable seeds, from when the cones are first recognizable until germination, which is relatively fast for a gymnosperm [41]. These characteristics make *Ephedra* attractive as a possible model species; however, the disadvantage is that it also has one of the largest genomes known among gymnosperms (i.e., 8.09–38.34 pg/1C) [2, 3, 42–45].

Here, we studied the different seed morphologies with a particular focus on the bracts of two species of *Ephedra* (Fig. 1) with the aim of: (i) detecting the genes involved in bract development; (ii) detect similarities and differences in gene expression; and (iii) generate fundamental molecular information for members of the genus with the greatest potential to become a model gymnosperm species. We used RNAseq, a methodology known for its efficiency in generating large-scale molecular information to address questions about non-model species [46] and a candidate gene approach. We present major morpho-anatomical and genetic differences found between the two seed morphologies studied here: *Ephedra californica* and *Ephedra antisyphilitica* (Fig. 1). Our results include the identification of differentially expressed (DE) genes in these two species (Fig. 1).

## Results

### Transcriptome assembly statistics

This study focused on the genes specific to the ovule and surrounding structures (bracts). De novo reference transcriptomes of *Ephedra californica* and *Ephedra antisyphilitica* were generated from total RNA isolated from bracts, young ovulate cones, ovules without bracts, pollen cones and shoots.

The total RNA of the different tissues was sequenced separately to identify the genes expressed in the bracts characterized by different morphologies, dry for *Ephedra californica* and fleshy bracts for *Ephedra antisyphilitica* (Additional file 1: Figs. S1, S2). Using Trinity software, 64,263 transcripts were obtained for *Ephedra californica* and with an average GC content of 41.14% (Table 1). Based on read coverage, the E90N50 statistic was 1.4Kb (Additional file 1: Fig. S3), the reference transcriptome contained 84.9% of the conserved Embryophyte genes using BUSCO annotation (Additional file 1: Fig. S4). For *Ephedra antisyphilitica*, 59,002 transcripts were obtained, with an average GC content of 41.2% and with a maximum assembled contig length of 13,825 (Table 2). Based on read coverage, the E90N50 statistic was 1.4Kb (Additional file 1: Fig. S3) and the reference transcriptome contained 87.8% of the conserved Embryophyte genes using BUSCO annotation (Additional file 1: Fig. S4).

**Table 1:** *Ephedra californica* reference transcriptome statistics

Parameter	Number
Total trinity transcripts	64,263
Total trinity 'genes'	27,958
Average 'genes' length (bp)	1931
%GC	41.14
Number of contigs > 200 bp	64,263
Number of contigs > 1 Kb	35,518
Number of contigs > 5 kb	553
Number of contigs > 10 Kb	6
Number of predict ORFs (transdecoder)	61,200

**Table 2:** *Ephedra antisyphilitica* reference transcriptome statistics

Parameter	Number
Total trinity transcripts	59,002
Total trinity 'genes'	27,095
Average 'genes' length (bp)	1967
% GC	41.2
Longest contig (bp)	13,825
Shortest contig	201
Number of contigs > 200 bp	59,002
Number of contigs > 1 Kb	32,891
Number of contigs > 5 kb	750
Number of contigs > 10 Kb	23
Number of predict ORFs (transdecoder)	55,100

**Fig. 2:**
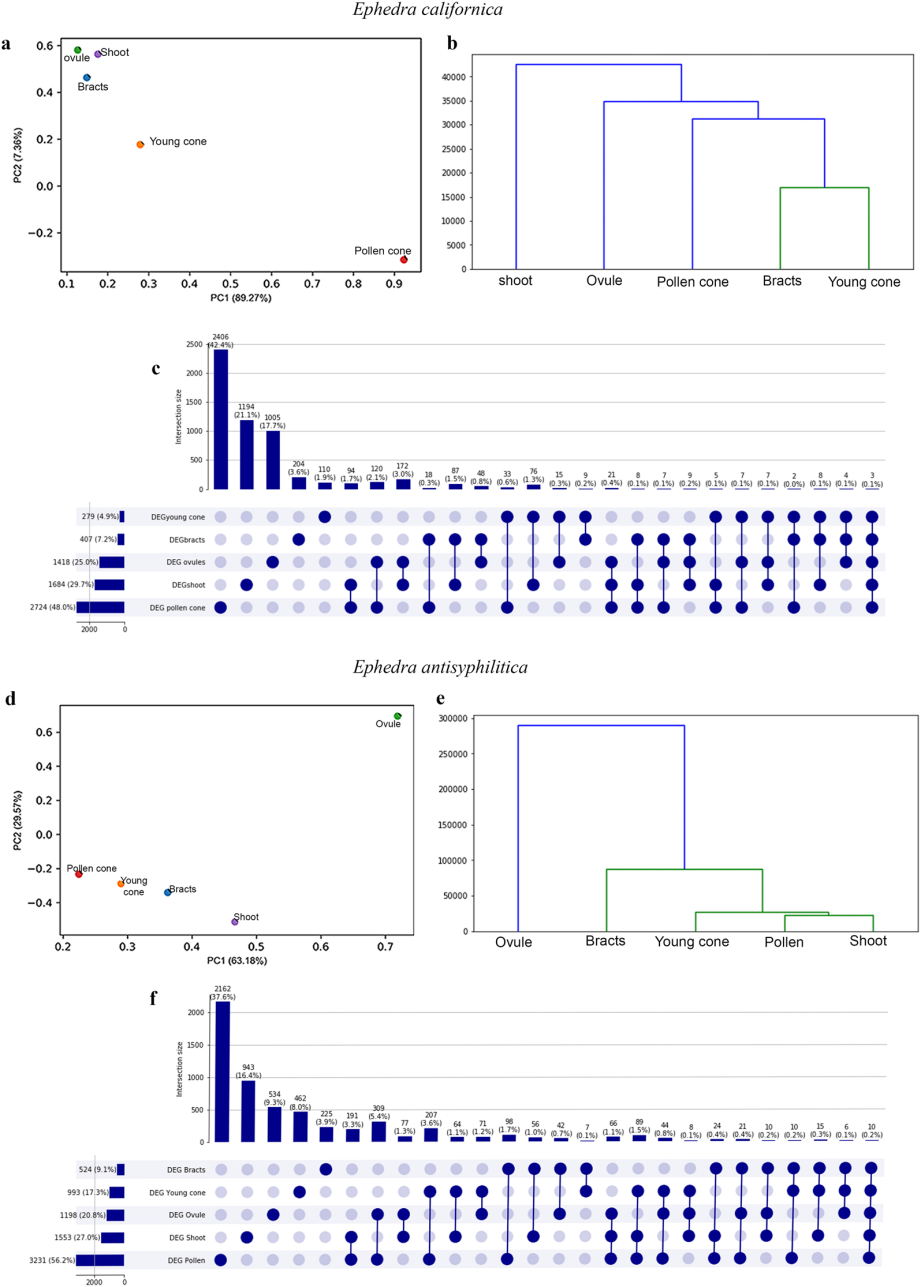
Comparisons between the different organ samples in each species. **a**, **b** *Ephedra californica.* **a** PCA and **b** Hierarchical clustering. **c** UpSet plot showing shared and unique DE genes for all tissues in *E. californica*, as an alternative to a Venn diagram. Each bar corresponds to a set, and on top is shown the size of the set, in number of genes and percentage. Filled-in circles at the bottom show which set is part of an intersection, lines connecting the filled-in cells show the intersection between the groups. In *E. californica*, 3 genes (0.1%) are shared between all the samples. **d** *Ephedra antisyphilitica* PCA, **e** Hierarchical clustering. **f** UpSet plot showing shared and unique DE genes for all tissues in *E. antisyphilitica,* 10 genes (0.2%) are shared between all the samples

Using a PCA method and a hierarchical clustering dendrogram, an initial comparison among *Ephedra californica* samples of the ovule and the pollen cone tissues shows major differences in terms of gene expression levels (Fig. 2a; Additional file 1: Fig. S5). Subsequently, the hierarchical clustering shows that the bracts and the young ovulate cones share similar gene expression levels, forming a cluster; whereas shoot, ovule and the pollen cone show greater distances and form another cluster, stressing their differences in gene expression levels (*Y*-axis; Fig. 2b). With an UpSet plot, we have graphed the genes that co-occur or that are mutually exclusive in the different samples, similarly to what is shown with a Venn diagram but facilitating the visualization for a large number of sets as in our case [47]. With the UpSet plot it is possible to observe that only three genes are shared between all the samples, corresponding to a 0.1% and that ovules and shoots have 172 genes in common, 3% (Fig. 2c). The three shared genes throughout the tissues of *E. californica* correspond to a Calmodulin-like protein which is a primary calcium sensor and in plants convert calcium signals into transcriptional responses regulating plant development and stress [48]. And also, two 4-coumarate-CoA ligase like 1, an enzyme which, interestingly, in Arabidopsis is mostly restricted to the tapetum [49].

The analysis of *Ephedra antisyphilitica* shows a different pattern. In this species, the levels of gene expression in the ovule are largely different from the expression levels found in the pollen cone, shoot, young ovulate cone and bracts, which, together form one cluster (*Y*-axis; Fig. 2d,e). The UpSet plot for all the samples in *E. antisyphilitica*, shows that there are 10 genes in common between all the samples (Fig. 2f). Including several for which no annotation has been retrieved; an ATP phosphoribosyltransferase 2, chloroplastic which is involved in amino acid biosynthesis; and an E3 ubiquitin-protein ligase RFI2-like which in involved in seedling development like hypocotyl elongation, it also regulates genes involved in flowering transition all these roles are involved with a photoperiodic response [50].

### Specific search for AGAMOUS, AGL6 and B-sister gene homologs

**Fig. 3:**
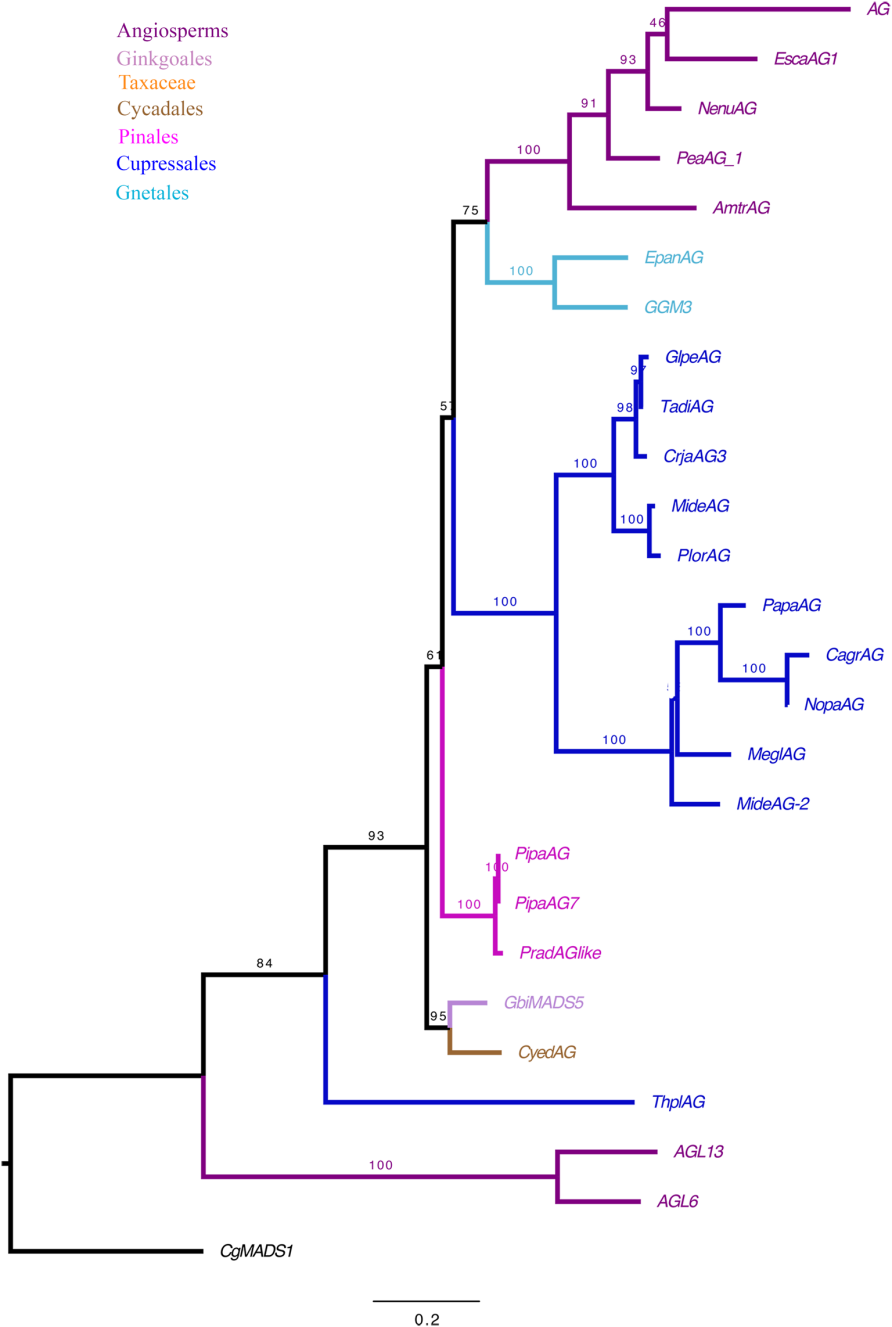
Maximum Likelihood analysis of *AGAMOUS,* involved in ovule development with emphasis on gymnosperms. Bootstrap (BS) values higher than 60 are shown on top of the branches. Colors follow the top left key. Names of the sequences, unless previously assigned, were assigned here using the two first letters of the genus and species followed by the gene family name (i.e., *Thuja plicata AG* homolog: *ThplAG*)

*AG, B-sister* and *AGL6* genes belong to the well-known MADS-box transcription factor family, for which the evolution has been well studied in angiosperms [51]. For this study, the maximum likelihood (ML) analyses presented here focused on gymnosperms. The phylogeny of the *AG* gene lineage was performed with 25 sequences, including 18 from gymnosperms and 7 from angiosperms; with no major duplication event identified in this gene lineage (Fig. 3). While no *AG* homolog was retrieved for *Ephedra californica*, one homolog was found in *Ephedra antisyphilitica*.

**Fig. 4:**
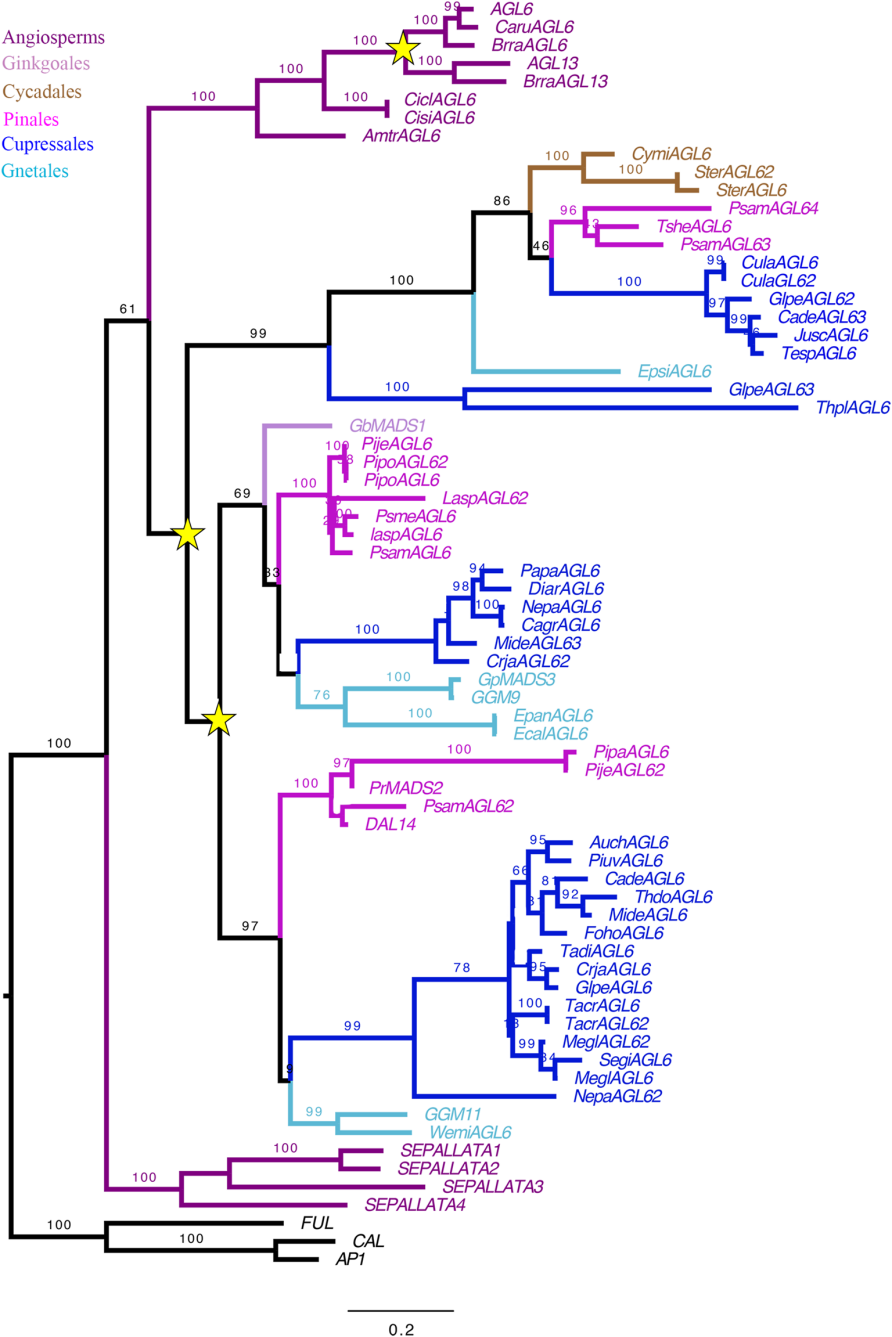
Maximum Likelihood analysis of the *AGL6* gene lineage, known to be involved in ovule development. Bootstrap (BS) values higher than 60 are shown on top of the branches. Yellow stars indicate major duplication events; colors in the tree follow the top left key. Names of the sequences, unless previously assigned, were assigned using the two first letters of the genus and species followed by the gene family name (i.e., *Amborella trichopoda AGL6* homolog: *AmtrAGL6*)

The *AGL6* phylogenetic hypothesis includes 67 sequences, 55 from gymnosperms and 12 from angiosperms, with two major duplication events detected (Fig. 4). One specific to Brassicaceae giving rise to *AGL6* and *AGL13* [16] and two duplication events that seems to have predated the diversification of gymnosperms [52]. However, since the homologs of *Ginkgo* and Cycadales are only found in one clade it is difficult to trace exactly when the duplication occurred (Fig. 4). Finally, homologs of the two *Ephedra* species, *EpanAGL6* and *EcalAGL6*, were retrieved.

**Fig. 5:**
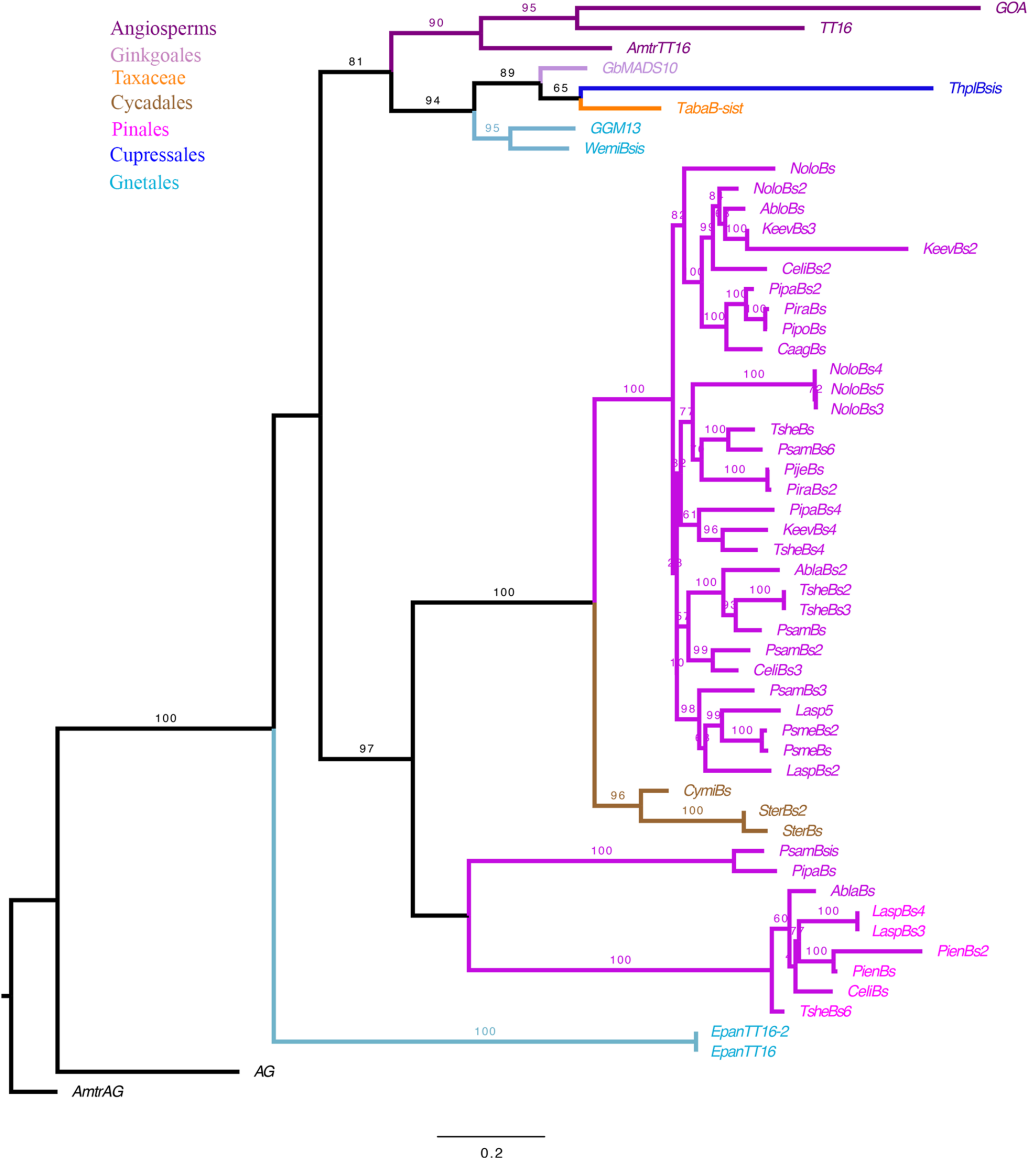
Maximum Likelihood analysis of the *B-sister* genes: *TRANSPARENT TESTA 16* (*TT16*) and *GORDITA* (*GOA*). Colors in the tree follow the top left key. Bootstrap (BS) values higher than 60 are shown on top of branches. Names of the sequences, unless previously assigned, were assigned using the two first letters of the genus and species followed by the gene family name (i.e., *Taxus baccata B-sister* homolog: *TabaB-sis*)

ML analysis of *B-sister* genes was performed with 3 angiosperm sequences and 49 gymnosperm sequences (Fig. 5). This clade includes the Brassicaceae specific clades [20]: *GORDITA* (*GOA*) and *TT16* (also known as, *Arabidopsis B sister*, *ABS*). A thorough BLAST was performed looking for homologs in *Ephedra*, but only two copies were retrieved from *E. antisyphilitica* and none from *E. californica* (Fig. 5).

### Differentially expressed genes in *Ephedra californica* and *Ephedra antisyphilitica* tissues

**Fig. 6:**
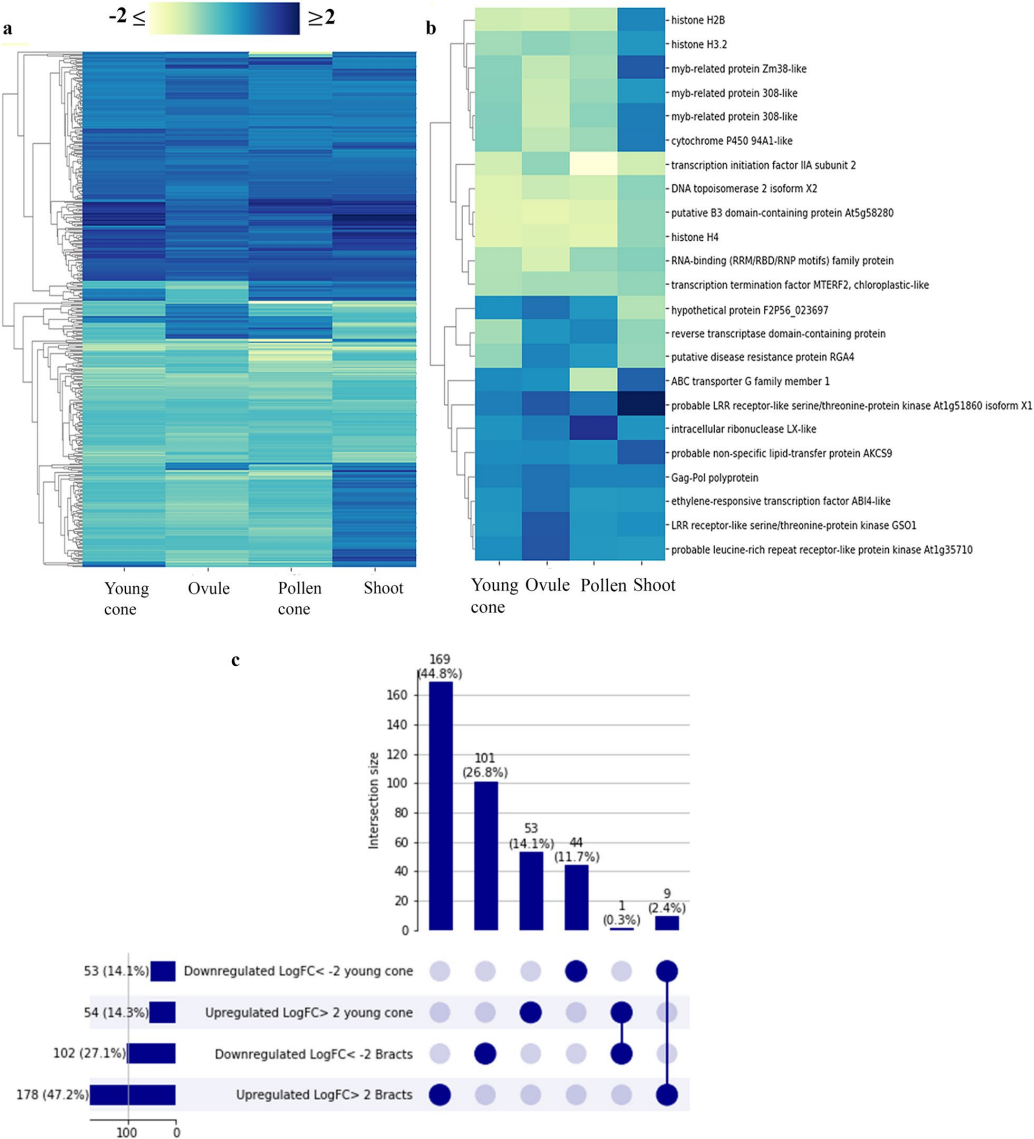
*Ephedra californica* transcriptome analyses. **a** Cluster map of differentially expressed (DE) genes with a ≥ twofold expression change and good transcriptional support (TPM ≥ 0.95) were considered (*n* = 407). Each column of the heatmap indicated the twofold changes of each sample with respect to the bract. In blue are upregulated genes and yellow downregulated genes. **b** Cluster map of only DE coding regions DE. **c** UpSet plot comparing DE genes up- and down- regulated between young ovulate cones and bracts. This representation is an alternative to a Venn diagram allowing for a better visualization of the sets; each bar is a set showing its size (in number of DE genes and percentage); filled-in circles at the bottom show which set is part of an intersection, lines connecting the filled-in cells show the intersection between the groups. Interestingly, 2.4% of shared genes between young cones and bracts are downregulated in the former and upregulated in the later

**Fig. 7:**
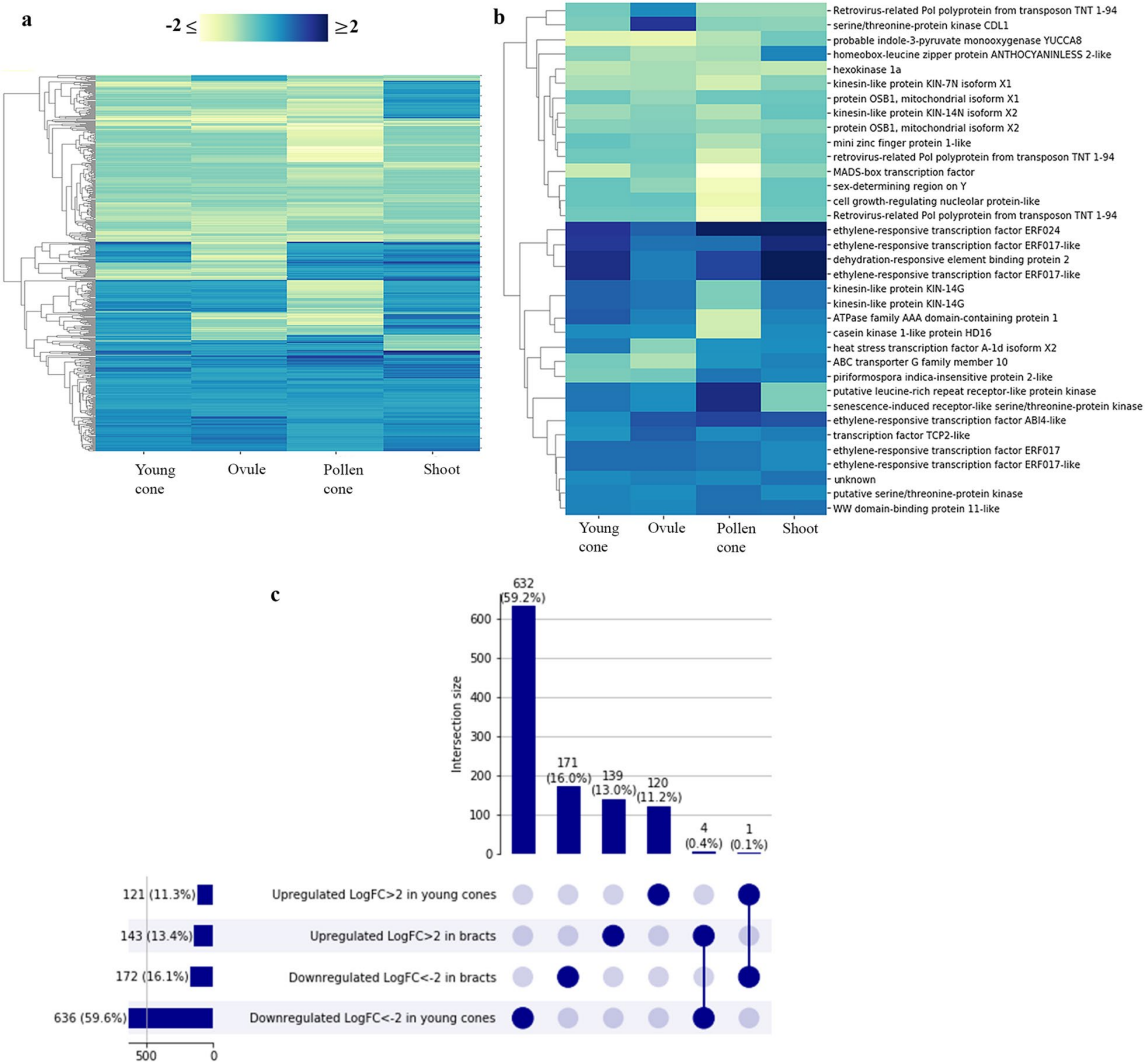
*Ephedra antisyphilitica* transcriptome analyses. **a** Cluster map of DE genes with a ≥ twofold expression change and good transcriptional support (TPM ≥ 0.95) were considered (*n* = 524). Each column of the heatmap indicated the twofold changes of each sample with respect to the bract. In blue are upregulated genes and yellow downregulated genes. **b** Cluster map of only coding regions DE. **c** UpSet plot comparing DE genes up- and down- regulated between young ovulate cones and bracts. This alternative to a Venn diagram allows for better visualization of the sets; each bar is a set showing its size (in number of DE genes and percentage); filled-in circles at the bottom show which set is part of an intersection, lines connecting the filled-in cells show the intersection between the groups

Identification of differentially expressed (DE) genes in the bracts of *Ephedra species*, that possibly play a significant role in their identity and their morphological differentiation, was carried out through transcriptome analyses in different plant tissues (i.e., bracts, young ovulate cones, ovules dissected, pollen cones and shoots), with three biological replicates (Additional file 1: Fig. S1). DE genes were filtered by statistical significance (FDR *p* ≤ 0.05), followed by a comparison of all tissues against bracts, since the focus is on the development of the bracts. Subsequently, to reveal the genes with a larger change and to identify genes with major differences in the expression, a fold change threshold was added (log2FC ≤ − 2 and ≥ 2), detecting 407 DE genes in the bracts of *Ephedra californica* and 524 DE genes in the bracts of *Ephedra antisyphilitica* (Figs. 6a, 7a).

### DE in the dry bracts of *Ephedra californica*

To assign homology, all DE genes were subjected to a gene ontology (GO) enrichment analysis using Blast2GO (www.blast2go.com/). Many genes that have a large impact on development, encode transcription factors, proteins involved in signaling, and cell division. Hence, we mainly looked for genes falling under those categories, according to the GO and we found that in *Ephedra californica*, there are 23 DE genes and the differential expression of each of them within tissues was also compared (Fig. 6b; Additional file 2: Table S1). Of these coding regions, compared to other tissues, seven are found to be largely upregulated in the bracts (Additional file 2: Table S1). These up-regulated genes include: two LRR receptor-like serine/threonine-protein kinase, one like *GSO1* (similar to At4g20140) and the other similar to At1g51860 in Arabidopsis; a leucine-rich repeat receptor-like kinase (similar to At1g35710 in Arabidopsis); one *ETHYLENE RESPONSIVE TRANSCRIPTION FACTOR* (*ERF*, similar to At2g40220); one intracellular ribonuclease LX-like; a non-specific lipid transfer protein AKCS9; and a Gag-Pol polyprotein. In addition, compared to all the other tissues, in the integument there are 16 downregulated genes. Among the DE genes, there are three histones (H2B, H3.2, H4) and three putative MYB-related proteins (one Zm38-like and two 308-like; Additional file 2: Table S1).

To identify genes involved in the early development of the bracts, a comparison was made among the genes differentially expressed in the ‘young ovulate cone’ sample, including young ovules and early developing bracts, and the sample named ‘bracts’ which corresponds to a later stage in development when the bracts cover the entire longitude of the seed (older bracts). In *Ephedra californica* 26 genes were found in both tissues (Fig. 6c). Among the shared genes found, there are nine uncharacterized sugar transport proteins, abscisic acid hydrolases; these genes are downregulated in the young cone and upregulated in the bracts (Fig. 6c). The *AGL6* homolog and another MADS-box gene likely to be an *AGL18* homolog (Additional file 3: Table S2), is upregulated in the young cone and downregulated in the bracts (Fig. 6c).

### DE in the fleshy bracts of *Ephedra antisyphilitica*

Similarly, in *Ephedra antisyphilitica*, all 524 DE genes were annotated to identify gene ontologies (GO) using Blast2GO (www.blast2go.com/). Focusing on identifying genes that mainly encode transcription factors, proteins involved in signaling, and cell division according to the GO categories, 34 were detected (Additional file 4: Table S3). From which, 10 are upregulated, which include six putative members of the DREB subfamily within the large ethylene-responsive transcriptome factor family (five similar to ERF017, At1g19210; and one similar to At2g40220); one putative serine/threonine-protein kinase; one TCP2-like; one WW domain-binding protein 11-like and one unknown (Fig. 7b; Additional file 4: Table S3).

The shared between the ‘young ovulate cones’ sample and the ‘bracts’ sample may allow the identification of genes involved in bract development from early stages (Fig. 7c). Among these, there are sequences of unknown function, and ethylene-responsive transcription factors, R2R3-MYB-like genes, and a *TT16* MADS-box transcription factor (Additional file 5: Table S4).

## Discussion

The main interest of this study is to determine the genetic differences involved in the development of two bract morphologies, dry membranous bracts in *Ephedra californica* and fleshy bracts in *Ephedra antisyphilitica* [4, 5]. It should be noted that in addition to exhibiting notable differences from other seed plants, *Ephedra* lacks transcriptomic and genomic data that are mostly available for angiosperms. Therefore, to draw a conclusion on the subject, it is necessary to perform further expression analyses and functional characterization of the genes detected here.

### Major differences between seed developmental stages detected by overall comparisons of gene expression level

To better visualize the changes that occurred in gene expression levels as the seed matures, and to filter the large datasets obtained for the two *Ephedra* species, we used a PCA analysis and the hierarchical clustering dendrogram, which revealed a clear difference in the gene expression levels in the ovule at an early stage of development and that of the mature ovule in both species [53, 54] (Fig. 2).

The young ovulate cone, on the other hand, has bracts in an early developing stage and young ovules, which would explain the similarities between the genes expressed in the bracts and in the young ovulate cones. This could suggest that the bract regulatory network is maintained throughout its development (Fig. 2b, d). Interestingly, in *E. antisyphilitica*, the regulatory network in the ovule appears to be completely different from that shown in bracts and young ovulate cones, which seem to share similarities in the gene expression levels with the shoots and pollen cones (Fig. 2c, d).

### Candidate genes for fleshy-seed development: AGAMOUS, AGL6 and B-sister genes, in *Ephedra* species

MADS-box genes have been broadly studied and their functions range from root development to floral transition, to specification of floral organ specification to fruit development [18, 24, 51, 55–57]. Of particular interest are the MADS-box genes: *AGAMOUS, AGL6* and *B-sister* genes, which were initially characterized for their role in the development of carpel and reproductive structures in gymnosperms and are also known for their role in ovule development in seed plants [14, 20, 21, 26, 28, 29, 58]. Several studies have assessed the expression of *AG* homologs in gymnosperms, focusing mainly on the species that develop fleshy seeds. In *Cycas*, *AG* is expressed in the outer layers of the integument (sarcotesta; [14]). In *Ginkgo* and *Taxus*, *AG* homologs are expressed throughout the ovule and seed including the aril [16]. In *Gnetum*, the *AG* homolog, *GGM3*, is found expressed throughout the strobilus, including the ovule, envelopes (integument and bracts), and pollen cones [19]. For this study, an extensive BLAST search was performed in the generated *Ephedra* transcriptomes, revealing homologs for *E. antisyphilitica*. However, no *AG* homolog was retrieved for *Ephedra californica* (Fig. 3).

*AGL6* is the sister clade of *SEPALLATA* (*SEP*), and unlike the *SEP* genes, *AGL6* has homologs across seed plants, which are expressed in the reproductive structures [21, 26]. In *Ginkgo* and *Taxus*, the expression of *AGL6* is in the entire ovule, including the fleshy structures [16]. However, in *Gnetum* the same expression patterns are not found, where homologs are expressed in the pollen strobili (cones) and the nucellus [19, 26]. Our phylogenetic analyses of *AGL6* in gymnosperms have shown a previously identified gymnosperm-specific duplication event [52] (Fig. 4). It is complex to trace exactly when this duplication occurred because a single clade contains representative sequences of *Ginkgo*, *Cycads* and *Ephedra*. However, a gene duplication event may involve a diversification of the function of these genes [59–61]; which makes it necessary to continue studies on these homologs of gymnosperms.

In angiosperms, the *B-sister* genes have been shown to be involved in the development of the seed coat, but the two paralogs of *Arabidopsis TT16* and *GOA*, have different functions [28, 32, 33, 62, 63]. Whereas *TT16* functions in the endothelium (the inner layer of the seed coat), *GOA* functions in the outer layer of the seed coat following a neo-functionalization event [20, 32]. In addition, these genes are involved in the expansion of fruit cells [32, 64]. Expression studies in gymnosperms show differences between species. In *Ginkgo*, for instance, the *TT16* homolog is expressed throughout ovule development and a role in seed ripening has been suggested. However, it does not seem to be involved in the development of the fleshy aril of *Taxus* [17] nor in the fleshy seed of *Gnetum*. The *Gnetum* homologs, *GGM2* and *GGM15*, are found expressed in the pollen strobili [19]. It is important to highlight that the integument, seed coat, is fleshy in *Ginkgo*, whereas in *Taxus* and *Gnetum* the aril and envelopes, respectively, which are additional structures covering the seed, are fleshy [65–68]. This suggests that *TT16* function is in the development of the ovule itself rather than in the fleshy characteristic. To better understand the role of these genes during the development of the reproductive organ but also to determine their involvement in the fleshy characteristic of these seeds, functional studies would be necessary.

Interestingly, we did not find homologs of *AG* and *TT16* in *Ephedra californica*, species with dry seeds. Several factors could explain the absence of these genes in *Ephedra californica*: (1) the expression levels are very low and therefore, more in-depth sequencing would be required to detect it; (2) that *AG* and *B-sister* are expressed in tissues or organs different to those from which transcriptomes were generated; and (3) that there is a true gene loss, which is difficult to assess, until more genomes become available (Figs. 3, 5).

### Key differences in gene regulation between vegetative (shoot) and reproductive tissues, including bracts, in *Ephedra californica*

In *Ephedra*, the leaves are extremely reduced and the shoot is therefore the main photosynthetic organ of the plant [4, 5, 65, 69]. In spite of that, heatmaps analyses in *Ephedra californica*, show significant differences in gene expression levels between the shoot and the other tissues (Fig. 6).

In terms of DE genes, several histone homologs are strongly up-regulated in the bracts compared to the shoot, such as histones H2B and H3 (Fig. 6b). These histones, like others, are involved in chromatin structure of eukaryotic cells and are susceptible to post-transcriptional regulation [67–69, 102, 103]. Histone H4, which is important to give structure to the DNA by forming a heterotetramer with H3, is curiously downregulated [70]. H4 is a canonical histone expressed during synthesis (S) phase of the cell cycle. H2A, H2B and H3, on the other hand, are expressed during all the phases of the cell cycle, suggesting that the bract cells were not in active cell division at the time of collection [71, 72]. In addition, seed development in *Arabidopsis* is a coordinated process that requires crosstalk between the endosperm (nutritive tissue) and the seed coat; the epigenetic regulation of seed coat development plays a key role in this process (reviewed in Refs. [73, 104]). For example, the proteins of the Polycomb Group (PcG) are involved in seed coat development and arrest until fertilization, in a dosage-sensitive manner [74]. The Polycomb Repressive Complex 2 (PRC2) represses target loci by the deposition of trimethyl groups on lysine 27 of histone H3 [75–77]. It is interesting that several histones are upregulated in reproductive tissues of *Ephedra californica*, a species with relatively rapid development cycle for a gymnosperm, taking only a few months for the seed to fully develop, suggesting that PcG play a role in seed development timing in *Ephedra* [41]. Further studies using different techniques on *Ephedra* are still required to better understand how the seeds develop in this group of plants.

Furthermore, several MYB-related proteins 308-like are also upregulated in the bract compared to the shoot. These proteins are known in *Antirrhinum majus* to repress Phenylpropanoid and lignin biosynthesis [78] (Fig. 6b). Thus, downregulation of MYB-related proteins 308-like in the shoot, is most likely responsible for its strong lignification. Of particular interest are the proteins that are upregulated in the bracts compared to the other tissues (blue cluster; Fig. 6b). There are several serine/threonine-protein kinases that are upregulated, including some putative LRR-receptor-like, GS01 and GS02, which together are required during the development of the epidermal surface in embryos and cotyledons [79]. To make a better assessment of their putative function in distantly related species like *Ephedra*, it is essential to have more information on these proteins outside model species.

### AGL6-like and other MADS-box transcription factors among the 26 genes putatively expressed throughout dry bract development

In this study, we identified genes likely involved in bract development from early developmental stages, genes shared by young ovulate cones (including early stages of the bracts) and adult bracts were identified (Fig. 6c). Among the genes found, some structural genes have been identified here such as 60S ribosomal proteins L8 and L12, proteins involved in catabolic processes such as RRP6-Like 3 (https://www.uniprot.org/uniprot/A9LLI8); one *AGL6-like* homolog, and another putative MADS-box gene, suggesting that MADS-box genes play a key role in bract development (Additional file 2: Table S1).

### Several proteins containing an AP2-domain putatively involved in fleshy bract development of *Ephedra antisyphilitica*

Among the 597 differentially expressed genes, there are several differences in the level of gene regulation among tissues, which is evident in the heat map (Fig. 7). Only in the bracts of *Ephedra antisyphilitica* there are important upregulated genes (blue clusters, Fig. 7b). Dehydration-responsive element-binding protein 2 (DREB2), is a protein containing an AP2-domain as it is part of the DREB subfamily within the large APETALA2/ethylene-responsive element-binding protein [80]. Members of the DREB family are induced by abiotic and biotic stresses. Specifically, DREB2, which is highly upregulated in bracts, seems to be involved in improving tolerance and yield in cases of water limitation, in rice, for instance, this leads to a higher number of inflorescences [81]. This characteristic is key for a species that grows under very extreme conditions, in desert areas but also because the gene is upregulated in the bracts that protect the seed and will eventually become fleshy.

Other ERFs are also strongly upregulated in bracts, including ERF024 and several putative ERF017 homologs. It has been suggested that ERF024 and ERF017 proteins are involved in fruit ripening, as they have been identified in tomato, melon, and peach fruits at the time of maturation, using different genomic techniques [82–84]. Little is known about the function of these proteins, and their role in tissue ripening needs to be further explored, but it is likely that this function is conserved in several seed plant lineages as we have identified them upregulated only in the bracts of *Ephedra antisyphilitica* that become fleshy as the seed matures. Additionally, a putative *TCP2* homolog, a gene known in *Arabidopsis* for its role in the negative regulation of boundary-specific genes such as *CUC* [85] is upregulated (Fig. 7b). *TCP2* genes are also involved in development of the ovule [86]. To better understand the specific role that this gene may be playing in bract development in *Ephedra antisyphilitica* further studies are needed.

Among the genes shared by the young ovulate cone and the bracts, likely to play a role throughout *Ephedra antisyphilitica* bract development, several ERF genes have been detected, as well as members of the R2R3-MYB gene family, widely known for their control of plant secondary metabolism [87] (Additional file 4: Table S3).

### Differentially expressed genes in bracts with different morphologies, dry and fleshy

Through this study it was possible to identify 407 DE genes in the bracts of *Ephedra californica* (Fig. 6a) and 524 DE genes in the bracts of *Ephedra antisyphilitica* (Fig. 7a). While several different genes seem to be involved in the development of the dry bract in *Ephedra californica*, strikingly, several members of the *APETALA2/ERF* transcription factor family appear to be involved in the development of the fleshy bract of *E. antisyphilitica* (Fig. 7). It is important to highlight that many genes putatively playing a key role in the development of the bracts of the two species have not been annotated (Additional file 2: Table S1, Additional file 4: Table S3), which means that they have no similarities or detectable homologs in other lineages [88, 89]. Two factors could explain this, on the one hand, it could be due to the limited number of genomes currently available for gymnosperms and on the other hand that, these genes could be species specific, or taxonomically restricted genes [90–92]. Taxonomically restricted genes are important for the development of specific novelties, generating morphological diversity [90]. Thus, further studies to properly annotate these ‘orphan genes’ are important to understand the unique bract development in *Ephedra*.

## Conclusions

The additional seed-covering structures (bracts) in *Ephedra* have been a subject of interest to plant developmental biologists for their ecological and functional importance. The transcriptomes that we present here generate fundamental molecular information for the development of new model species [45]. Furthermore, the outcomes of this study provide a solid framework for future research aimed at improving our understanding of the genetic network underlying the development of seed structures, relevant to seed viability, endurance and survival:

1. It is likely that the ovule developmental function of MADS-box genes, *AGL6*, *AG* and *TT16*, is conserved across seed plants, and that is why their expression is detected in some gymnosperms with fleshy seeds. However, the availability of functional studies in gymnosperms are necessary to determine if they are involved in the fleshy characteristic.
2. Without functional studies, it is difficult to pinpoint exactly which genes are responsible for the fleshy and dry seed phenotypes. However, here we detected that there are major genetic differences between the two seed morphologies: fleshy and dry, among *Ephedra* species. The fleshy seeds of *Ephedra antisyphilitica,* for instance, have several ERF genes upregulated, which have been associated with fruit ripening.
3. Our results show that the bracts, the additional structures covering the seed of *Ephedra,* do not have genetic similarities with integuments, thus supporting the hypothesis that there is only one integument in Gnetales, in contrast to what has been suggested that *Ephedra* and other Gnetales have more than one integument.
4. To better assess the function of the genes detected in this study, expression analyses are still necessary. In addition, due to the lack of functional methodologies, in situ hybridization experiments remain the technique to determine when and where these genes function.

## Methods

### Collection of plant material for RNAseq, total-RNA extraction for *Ephedra* spp. and Illumina sequencing

The species studied here were collected in the field. *Ephedra californica* ovules and shoots were collected in RNA-later at the Rancho Santa Ana Botanical Garden (RSABG; collection number: 7842). Additional samples (biological replicates) of shoots, ovules and pollen cones, were collected in the field (voucher: United States, California, Whitewater, Whitewater Canyon Rd, on the road to the entrance to the preserve No. 15–17. February 2018, Zumajo-Cardona C. and Mayer R, NYBG). *Ephedra antisyphilitica* shoots, ovules and pollen cones were collected in liquid nitrogen in the field (voucher: United States, Texas, Palo Pinto Mountains State Park No 18–21. Zumajo-Cardona C., Vasco A., Bordelon A., and O’Kennon B, NYBG). A total of five different samples for each *Ephedra* species were processed for sequencing with three biological replicates each, and dissected into bracts, young ovule cones, ovules, pollen cones and shoots as the leaves are inconspicuous (total of 15 samples sequenced per species; Additional file 1: Fig. S1). The experiment was conducted to compare the different parts of the plant, to identify their differences, with special focus on the bracts surrounding the ovule. Tissue was ground in liquid nitrogen and total RNA was extracted using PureLink Plant RNA Kit with Plant isolation aid (ThermoFisher Scientific). The quality of the total RNA was assessed using a Qubit 2.0 (ThermoFisher Scientific) and an Agilent Technologies 2100 Bioanalyzer. High-quality total RNA was used for preparing transcriptome libraries (ratio A260/A280 ≈ 2 and RIN ≥ 8). RNA-Seq libraries were prepared using NEBNext Poly(A) mRNA Magnetic Isolation Module Library Prep Kit (New England Biolabs) and the resulting libraries were paired-end (PE) sequenced (2 × 150 bp) using an Illumina HiSeq2000. The average sequencing depth for each sample was 40 million reads (Additional file 1: Fig. S2).

### De novo transcriptome assembly and gene annotation in *Ephedra*

The quality of raw reads was assessed using FastQC (Additional file 1: Fig. S1). Sequence adapters and low-quality reads (Phred score < 5) were removed using Trimmomatic (V 0.36) with all the default parameters [93]. Reads were assembled using Trinity pipeline (V 2.8.4; [95]). A reference transcriptome was assembled using all contigs with length ≥ 200 nucleotides from all RNA samples. The quality of the transcriptome assembly was assessed based on the calculated E90N50 contig length. The reference transcriptome was annotated using DIAMOND [94]. Contigs were searched against bacterial and fungal databases, mainly associated with soil and plants, sequence databases compiled from UniProt (uniport.org) to identify possible contaminants. Sequences with an identity ≥ 50% were removed from the reference transcriptome (*Ephedra californica* *N* = 3405; *E. antisyphilitica* *N* = 3229). Transcriptome quality was assessed with contig length and BUSCO annotation and the resulting assembly was used for the following steps. The long open reading frames (ORF) were predicted using TransDecoder (v 3.0.0) software. For gene annotation, *Ephedra* contigs were searched against several land plant protein coding sequence databases (*Amborella trichopoda:* AMTR1.0 13333, *Arabidopsis thaliana*: TAIR10 3702, *Capsicum annuum*: ASM51225v2, *Ginkgo biloba*: NCBI:txid3311, *Gnetum montanum*: NCBI:txid3381, *Oryza sativa*: IRGSP-1.0, *Picea abies*: NCBI:txid3329, *Selaginella moellendorffii*: V1.0 88036, *Vitis vinifera*: 12X 29760; available through Ensembl and PLAZA for gymnosperms; Additional file 1: Fig. S1).

### Construction of phylogenetic trees of candidate genes putatively involved in development of fleshy tissues

*AGAMOUS, AGL6* and *B-sister* (*TT16* and *GORDITA*, *GOA*) sequences from Arabidopsis were used to perform the initial BLAST search (*AG* = At4g18960; *AGL6* = At2g45650; *AGL13* = At3g61120; *TT16* = At5g23260 and *GOA* = At1g31140). The search was focused on the gymnosperms from the OneKP database (https://db.cngb.org/onekp/) and the *Ephedra* transcriptomes generated here (these sequences will be deposited in NCBI GenBank). The sequences were compiled and kept in the open reading frame using AliView [96]. The nucleotide sequences were aligned with MAFFT using a gap penalty of 3.0, an offset value of 0.5 (https://mafft.cbrc.jp/alignment/software/; [105]). To determine the nucleotide substitution model that best fits these gene lineages we used jModelTest 2 [97], which identified the GTRGAMMA model as the best-fit model for all our datasets. Maximum likelihood (ML) phylogenetic analyses using the nucleotide sequences were performed using RaxML-HPC2 BlackBox [98] available on the CIPRES Science Gateway portal [99]. Bootstrapping was performed according to the default criteria in RaxML where the boot-strapping stopped after 200–600 replicates. The resulting tree was finally observed and edited using FigTree v1.4.2 (http://tree.bio.ed.ac.uk/software/). The outgroups used for the *AGL6/ AGL13* phylogeny were closely related genes from *Arabidopsis* (*FRUITFULL* = At5g60910; *APETALA1* = AT1G69120 and *CAULIFLOWER* = At1g26310). For *AG*, the outgroup used was an Algae, *Chara globularis*, MADS-box sequence (CgMADS1 = AB035567.1) and *AGL6*, *AGL13* from *Arabidopsis*. The outgroup used for the *B-sister* phylogeny is the *Arabidopsis AG* homolog.

### Transcriptome abundance (RSEM) and expression level analyses (EB- Seq)

Sequenced reads from the different plant tissues were aligned to the reference transcriptome using Bowtie2 [100] and RSEM (RNA-Seq by Expectation Maximization) was used to obtain estimates of transcript abundance for all transcripts [101]. The resulting expression levels are calculated in terms of Transcripts Per Million (TPM).

A principal component analysis (PCA), with normalized TMP values, was used as it preserves the global data structure by forming well-separated clusters, allowing to detect major differences between samples, but it can fail to preserve the similarities within the clusters. Thus, in addition, a hierarchical clustering analysis using a complete linkage method, provided a dendrogram showing the relation between samples according to the levels of gene expression. These analyses were executed in Python3 using the libraries: pandas, sklearn and scipy. UpSet plot was used to represent shared and unique number of genes in each sample, as with a large number of sets (> 3) it allows a better visualization than a Venn diagram [47]. This was implemented using UpSet plot and matplotlib libraries in Python3.

Differential gene expression levels were assessed with EBSeq, using median normalized data. Genes were considered to be statistically significant differentially expressed with a TPM ≥ 0.95 for at least one single tissue. Fold change (log2FC) was calculated for bracts in relation to the other tissues, and only genes with a large change were kept (log2FC ≤ − 2 and ≥ 2) and with an FDR *p* ≤ 0.05 (fold discovery rate). The differentially expressed genes were further analyzed with Blast2Go (v 5.2.5) to identify the corresponding Gene Ontology (GO) terms. Results were plotted using different Python libraries (i.e., Matplotlib, Seaborn; Additional file 1: Fig. S1).

## Supplementary Information

**Additional file 1: Fig. S1.** *Ephedra* pipeline. **a** *Ephedra californica* ovules and pollen cone. **b** *Ephedra antisyphilitica* ovules and pollen cone. **c** Bioinformatics pipeline used for the transcriptome data, divided into three main steps. **Fig. S2.** Total number of reads obtained in **a** *Ephedra californica* **b** *Ephedra antisyphilitica.* **Fig. S3.** E90N50 Statistics for **a** *Ephedra californica* and b) *Ephedra antisyphilitica.* **Fig. S4.** BUSCO analysis against the Embryophyta database in **a** *Ephedra californica* and **b** *Ephedra antisyphilitica*. More than 80% of the transcriptomes are completed for both species. **Fig. S5.** PCA analyses using all replicates. *Ephedra californica* at the top and *Ephedra antisyphilitica* at the bottom.

**Additional file 2: Table S1.** List of transcription factors up- and downregulated in *Ephedra californica.*

**Additional file 3: Table S2.** List of DE genes shared between the young ovules and bracts of *Ephedra californica*.

**Additional file 4: Table S3.** List of transcription factors up- and downregulated *in Ephedra antisyphilitica.*

**Additional file 5: Table S4.** List of DE genes shared between the young ovules and bracts of *Ephedra antisyphilitica*.

## Data Availability

All supporting data are available in the Additional files. Newly identified sequences are available through NCBI. Any additional data can be requested to the corresponding author.
