# Supplementary material for: Fleshy or dry: transcriptome analyses reveal the genetic mechanisms underlying bract development in Ephedra
Source: EvoDevo. 2022 Apr 27;13:10. doi: 10.1186/s13227-022-00195-4 (PMC9047513; doi:10.1186/s13227-022-00195-4)
Supplement: Supplementary file 1 — Additional file 1: Fig. S1. Ephedra pipeline. a Ephedra californica ovules and pollen cone. b Ephedra antisyphilitica ovules and pollen cone. c Bioinformatics pipeline used for the transcriptome data, divided into three main steps. Fig. S2. Total number of reads obtained in a Ephedra californica b Ephedra antisyphilitica. Fig. S3. E90N50 Statistics for a Ephedra californica and b) Ephedra antisyphilitica. Fig. S4. BUSCO analysis against the Embryophyta database in a Ephedra californica and b Ephedra antisyphilitica. More than 80% of the transcriptomes are completed for both species. Fig. S5. PCA analyses using all replicates. Ephedra californica at the top and Ephedra antisyphilitica at the bottom. [file 13227_2022_195_MOESM1_ESM.docx]

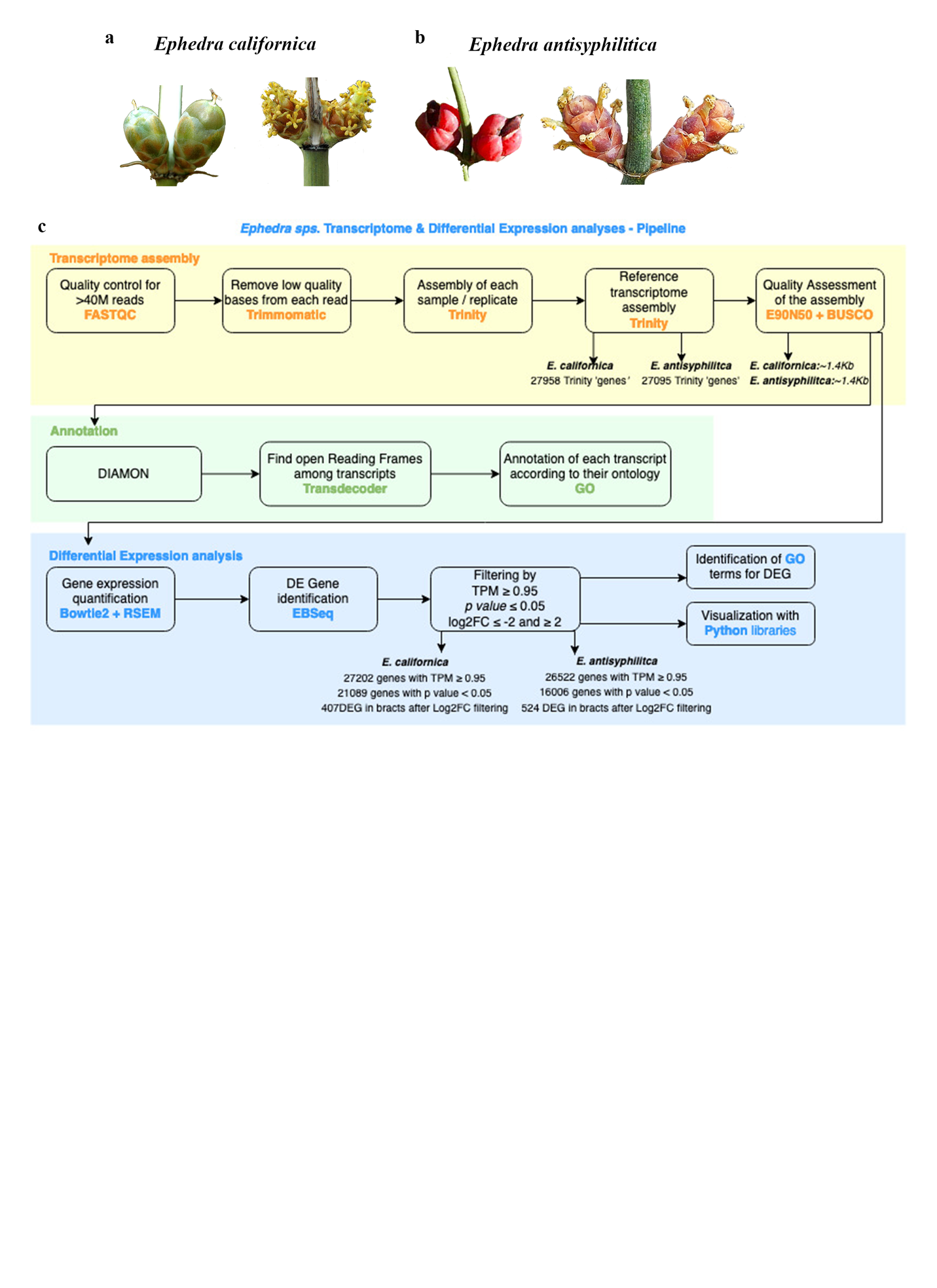


**Fig. S1.** *Ephedra* pipeline. **a.** *Ephedra californica* ovules and pollen cone. **b.** *Ephedra antisiphylitica* ovules and pollen cone. **c.** bioinformatics pipeline used for the transcriptome data, divided into three main steps.



**Fig. S2.** Total number of reads obtained in a) *Ephedra californica* b) *Ephedra antisyphilitica.*



**Fig. S3.** E90N50 Statistics for a) *Ephedra californica* and b) *Ephedra antisyphilitica.*



*
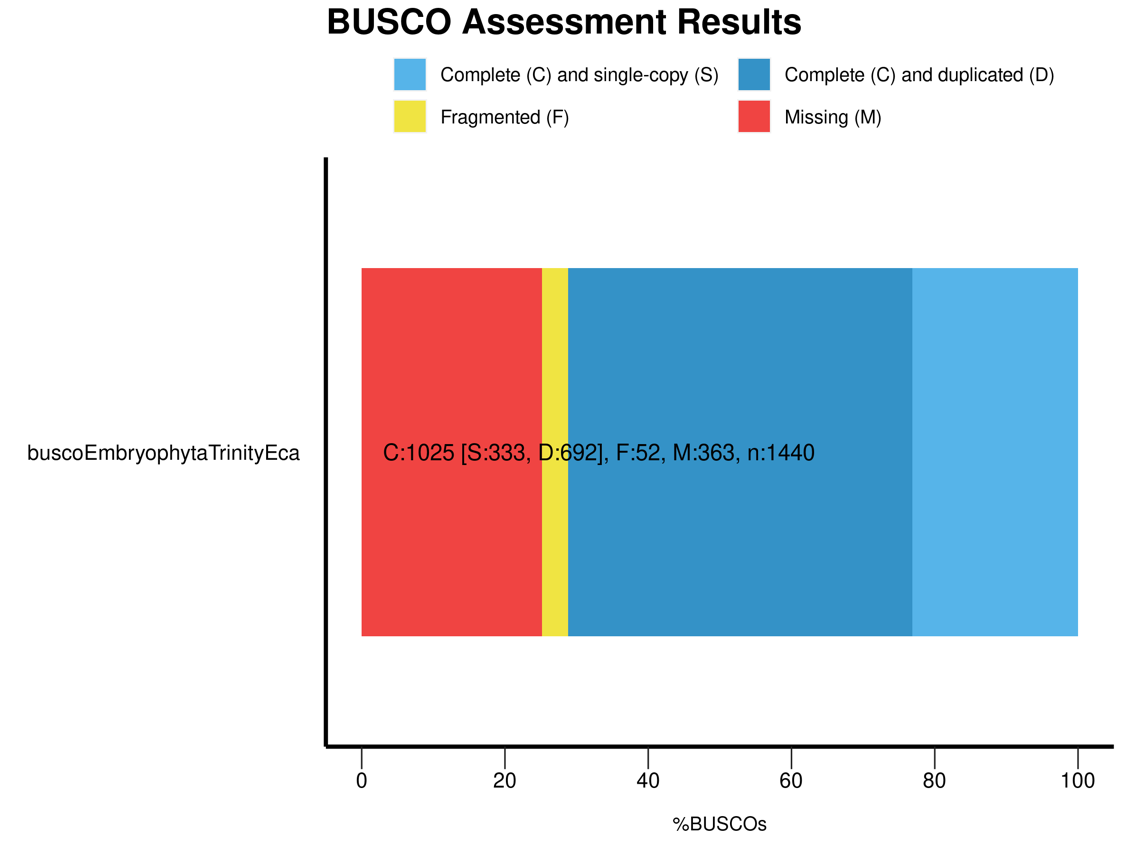

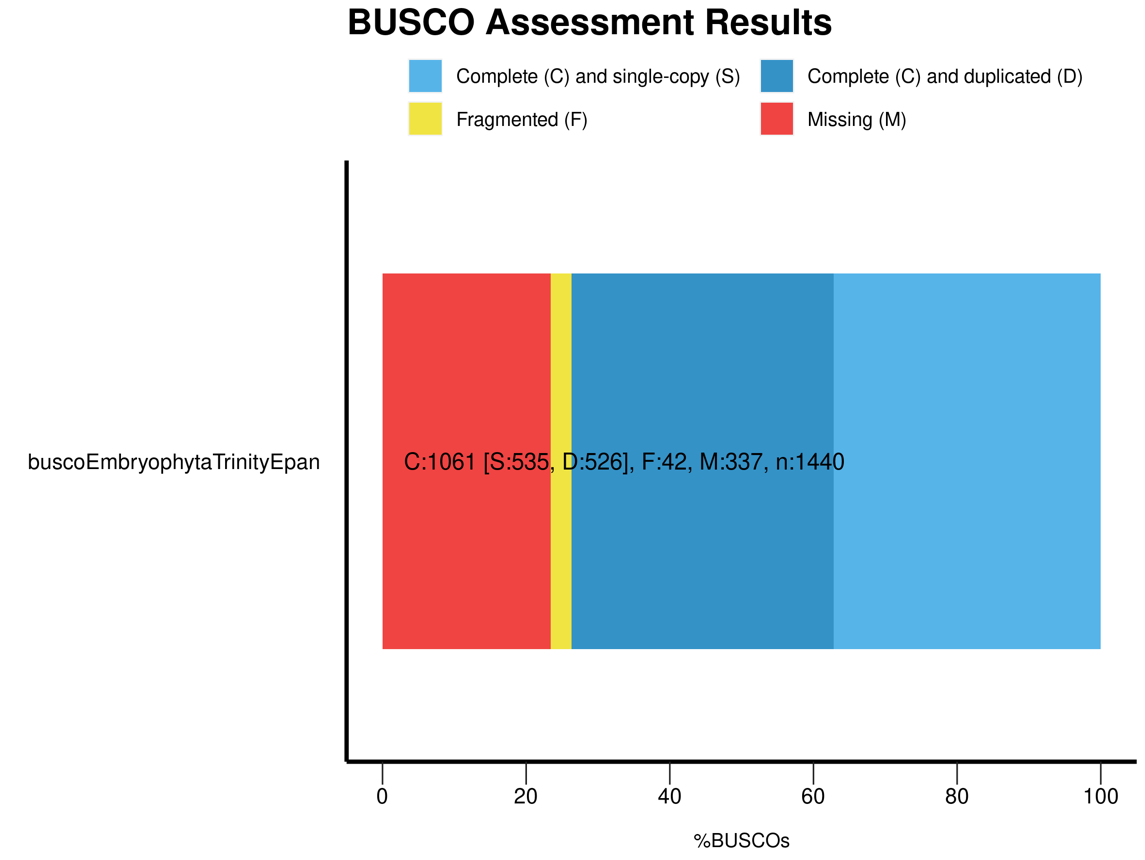
*



**Fig. S4.** BUSCO analysis against the Embryophyta database in a) *Ephedra californica* and b) *Ephedra antisyphilitica*. More than 80% of the transcriptomes are completed for both species.


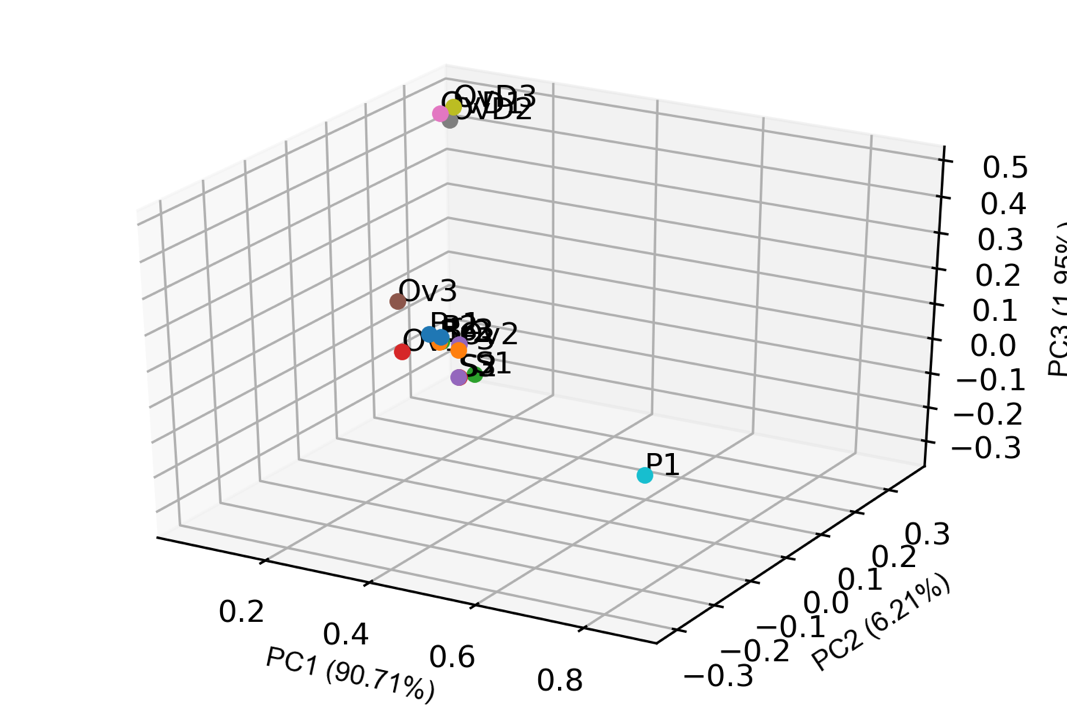


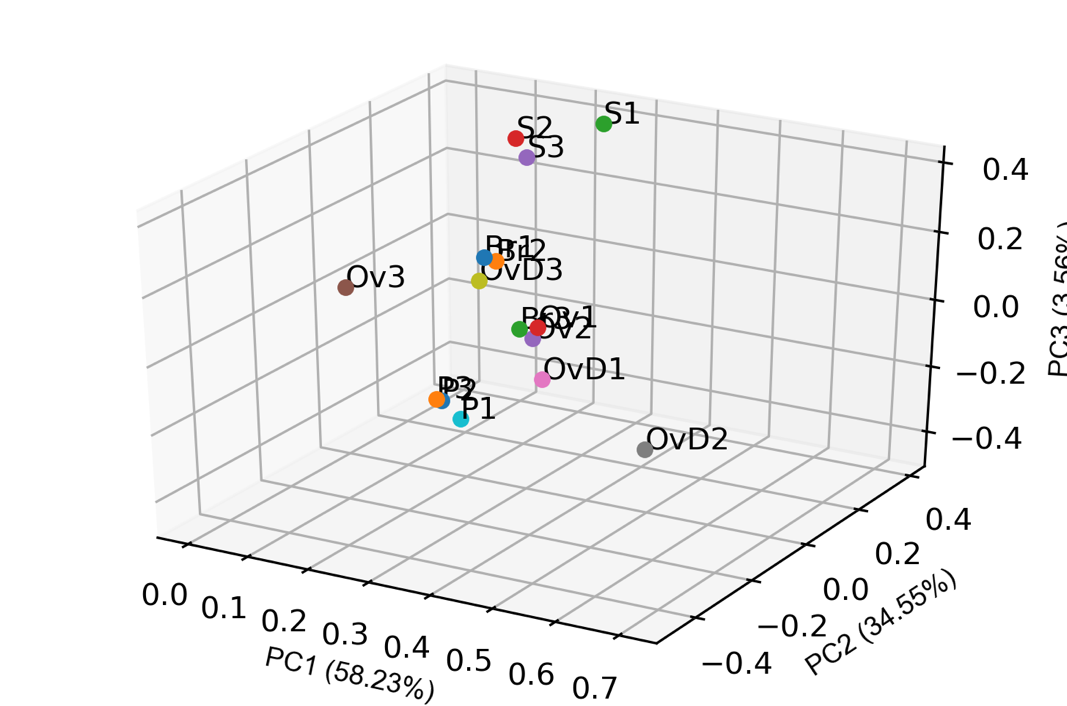


**Fig. S5.** PCA analyses using all replicates. *Ephedra californica* at the top and *Ephedra antisyphilitica* at the bottom.
